# Some Thoughts on Prostatectomy*Abstract of article in *New York Medical Journal*, September 9, 1916.

**Published:** 1916-11

**Authors:** Henry H. Morton

**Affiliations:** Clinical Professor of Genito-Urinary Diseases in the Long Island College Hospital; Genito-Urinary Surgeon to Long Island College and Kings County Hospitals and the Polhemus Memorial Clinic, Etc., Brooklyn, N. Y.


### "Some Thoughts on Prostatectomy."*

BY HENRY H. MORTON, M. D., CLINICAL PROFESSOR OF GENITO-URINARY DISEASES IN THE LONG ISLAND COLLEGE HOSPITAL; GENITO-URINARY SURGEON TO LONG ISLAND COLLEGE AND KINGS COUNTY HOSPITALS AND THE POLHEMUS MEMORIAL CLINIC, ETC., BROOKLYN, N. Y.

Cases of hypertrophied prostate may be divided into three groups:

1. Premonitory period, characterized by slight difficulty in starting stream, increased frequency, more marked at night, due to a passive congestion of the trigone and prostate and a reflex polyuria. During this stage there is no residual urine.

2. Stage of insufficiency, characterized by partial retention of urine and increased frequency, during day as well as night. This stage will continue on to one of complete retention.

3. Stage of incontinence, bladder becomes distended, sometimes up to the umbilicus, sphincter relaxing every few minutes, allowing a few drops of urine to escape.

Dilatation of the ureters, back pressure on the kidneys with a resulting cystitis, pyelitis and pyelonephritis come from this overdistended bladder, and the patient suffers from urosepsis, as shown by a slight temperature, a dry, brown tongue, stomach disturbances, a dry, scaly skin, and a sluggish mentality.

The diagnosis is made by rectal palpation of prostate, determination of amount of residual urine and a cystoscopic examination.

Before operation is decided upon the kidney function must be determined. The phenol sulphonephthalein test is the best. Where the function is low, it must always be brought up by continued preoperative treatment, which consists of permanent bladder drainage by means of a retained catheter; after the bladder has been

---

*Abstract of article in *New York Medical Journal*, September 9, 1916.

emptied slowly, daily irrigations of silver nitrate, 1-4000, till cystitis clears up, forced water and urinary antiseptics.

The two-stage operation is reserved for a few cases which become intolerant to the catheter, but it is to be condemned on account of the sloughing wound and the adhesions around the bladder.

Choice of operation: Where finger cannot reach the upper border of prostate or there is marked intravesical enlargement, suprapubic prostatectomy is indicated.

Where prostate is not much enlarged and placed low down, the perineal operation is chosen.

Postoperative treatment consists in the use of the Murphy drip and the electric baker till kidneys functionate. Water should be forced by mouth as soon as patient can swallow.

Tympanites is treated by strychnine and eserin, piturin or hormonal internally, and turpentine stupes over abdomen. Hiccough is controlled by the use of atropin. Stomach lavage is very useful where the vomiting is severe.

Hemorrhage at the time of operation is controlled by a firm tampon in the cavity left after prostate has been removed.

A dental rubber dam dressing helps collect the urine from a suprapubic wound and makes the patient a great deal more comfortable.

Mortality rate varies from 10 to 40 per cent. With good preoperative treatment it should be about 10 per cent.

At the Long Island College Hospital, in the past three years, forty-three operations; six deaths; one due to carcinoma of the stomach, four weeks postoperative; one due to cardiac trouble, six weeks postoperative; and four due directly to operation because of poor renal function.

H. V. RAYCROFT, M. D.

---

Dr. M. W. Wooten of San Antonio, Texas, has gone to New York to spend eight months in special study.

---

Dr. Chas. L. Gregory of Greenville, Texas, has recently remodeled and enlarged his sanitarium, Park View Retreat, and it is now one of the most complete and up-to-date institutions in that part of the country.

